# Functional connectivity associations with markers of disease progression in *GRN* mutation carriers

**DOI:** 10.1002/alz.087301

**Published:** 2025-01-03

**Authors:** Taru M. Flagan, Stephanie A. Chu, Suvi Häkkinen, Liwen Zhang, David McFall, Carolin Heller, Jonathan D. Rohrer, Jesse A. Brown, Alex Jihun Lee, Kristen Fernhoff, Lorenzo Pasquini, Maria Luisa Mandelli, Maria Luisa Gorno Tempini, Jennifer S. Yokoyama, Virginia Sturm, Brian Appleby, Brad C Dickerson, Kimiko Domoto‐Reilly, Tatiana M. Foroud, Daniel H. Geschwind, Nupur Ghoshal, Neill R Graff‐Radford, Murray Grossman, Ging‐Yuek Robin Hsiung, Eric J. Huang, Edward D. Huey, Kejal Kantarci, Anna M. Karydas, Daniel Kaufer, David S. Knopman, Irene Litvan, Ian R MacKenzie, Mario F. Mendez, Chiadi U. Onyike, Leonard Petrucelli, Eliana Marisa Ramos, Erik D Roberson, Julio C. Rojas, Maria Carmela Tartaglia, Arthur W. Toga, Sandra Weintraub, Leah K. Forsberg, Hilary W. Heuer, Brad F. Boeve, Adam L. Boxer, Howard J. Rosen, Bruce L. Miller, Fermin Moreno, William W. Seeley, Suzee E. Lee, the ARTFL/LEFFTDS Consortia

**Affiliations:** ^1^ University of California, San Francisco, San Francisco, CA USA; ^2^ Dementia Research Centre at UCL Queen Square Institute of Neurology, London United Kingdom; ^3^ Dementia Research Centre, Department of Neurodegenerative Disease, UCL Queen Square Institute of Neurology, University College London, London United Kingdom; ^4^ Memory & Aging Center, Department of Neurology, University of California in San Francisco, San Francisco, CA USA; ^5^ University of California San Francisco (UCSF), San Francisco, CA USA; ^6^ University of California San Francisco, San Francisco, CA USA; ^7^ Case Western Reserve University, Cleveland, OH USA; ^8^ Massachusetts General Hospital, Charlestown, MA USA; ^9^ University of Washington, Seattle, WA USA; ^10^ Indiana University School of Medicine, Indianapolis, IN USA; ^11^ David Geffen School of Medicine, University of California, Los Angeles, Los Angeles, CA USA; ^12^ Washington University School of Medicine, St. Louis, MO USA; ^13^ Department of Neurology, Mayo Clinic, Jacksonville, FL USA; ^14^ Frontotemporal Degeneration Center, Department of Neurology, Perelman School of Medicine, University of Pennsylvania, Philadelphia, PA USA; ^15^ University of British Columbia, Vancouver, BC Canada; ^16^ Brown University, Providence, RI USA; ^17^ Department of Radiology, Mayo Clinic, Rochester, MN USA; ^18^ University of North Carolina, Chapel Hill, NC USA; ^19^ Mayo Clinic, Rochester, MN USA; ^20^ University of California San Diego, La Jolla, CA USA; ^21^ The University of British Columbia, Vancouver, BC Canada; ^22^ UCLA David Geffen School of Medicine, Los Angeles, CA USA; ^23^ Johns Hopkins University School of Medicine, Baltimore, MD USA; ^24^ Mayo Clinic, Jacksonville, FL USA; ^25^ University of California, Los Angeles, Los Angeles, CA USA; ^26^ University of Alabama at Birmingham, Birmingham, AL USA; ^27^ Memory and Aging Center, Weill Institute for Neurosciences, University of California, San Francisco, San Francisco, CA USA; ^28^ Memory Clinic, Toronto Western Hospital, University Health Network, Toronto, ON Canada; ^29^ Laboratory of Neuro Imaging, Stevens Neuroimaging and Informatics Institute, Keck School of Medicine, University of Southern California, Los Angeles, CA USA; ^30^ Northwestern University Feinberg School of Medicine, Chicago, IL USA; ^31^ Department of Neurology, Mayo Clinic, Rochester, MN USA; ^32^ Memory and Aging Center, UCSF Weill Institute for Neurosciences, San Francisco, CA USA; ^33^ Hospital Universitario Donostia, San Sebastián Spain; ^34^ Memory and Aging Center, UCSF Weill Institute for Neurosciences, University of California San Francisco, San Francisco, CA USA

## Abstract

**Background:**

Autosomal dominant progranulin (*GRN)* mutations are a common genetic cause of frontotemporal lobar degeneration. Though clinical trials for *GRN*‐related therapies are underway, there is an unmet need for biomarkers that can predict symptom onset and track disease progression. We previously showed that presymptomatic *GRN* carriers exhibit thalamocortical hyperconnectivity that increases with age when they are presumably closer to symptom onset. However, whether hyperconnectivity arises concomitantly with markers of neurodegeneration remains unclear.

**Method:**

Utilizing T1 and task‐free functional magnetic resonance imaging (tf‐fMRI) from 49 presymptomatic and 26 symptomatic *GRN* mutation carriers, we determined the relationships between functional connectivity as measured by voxel‐wise whole brain degree and *GRN*‐relevant markers of disease progression, which included plasma neurofilament light chain (NfL) concentrations, CSF complement C1q and C3b protein levels, grey matter atrophy, and OCD symptom severity.

**Result:**

NfL concentrations were associated with frontotemporoparietal and thalamic hyperconnectivity in presymptomatic GRN carriers and extensive regions of atrophy in symptomatic carriers. Complement levels were associated with regions of hyperconnectivity, but not gray matter, in symptomatic carriers. Presymptomatic carriers with thalamic hyperconnectivity tended to have lower grey matter volume in bilateral insula and left lateral parietal cortex, which are among regions that deteriorate in *GRN*‐FTD. OCD symptom severity was associated with hypoconnectivity across all *GRN* carriers.

**Conclusion:**

In presymptomatic carriers, the co‐occurrence of hyperconnectivity, high NfL, and low gray matter suggests that tf‐fMRI hyperconnectivity may portend the onset of the neurodegenerative phase. These findings point toward hyperconnectivity as an indicator of approaching symptomatic onset.

